# A Comprehensive Study of the Effects by Sequence Truncation within Inverted Terminal Repeats (ITRs) on the Productivity, Genome Packaging, and Potency of AAV Vectors

**DOI:** 10.3390/microorganisms12020310

**Published:** 2024-02-01

**Authors:** Yinxing Chen, Shiliang Hu, William Lee, Noel Walsh, Kayla Iozza, Neil Huang, Gregory Preston, Lauren M. Drouin, Nannan Jia, June Deng, Matthias Hebben, Jing Liao

**Affiliations:** Genomic Medicine, Alexion, AstraZeneca Rare Disease, 65 Hayden Avenue, Lexington, MA 02421, USA; yinxing.chen@alexion.com (Y.C.); shiliang.hu@alexion.com (S.H.); william.lee@alexion.com (W.L.); noel.walsh@alexion.com (N.W.); kayla.iozza@alexion.com (K.I.); neil.huang@alexion.com (N.H.); gregory.preston@alexion.com (G.P.); lauren.drouin@alexion.com (L.M.D.); nannan.jia@alexion.com (N.J.); june.deng@alexion.com (J.D.); matthias_hebben@hotmail.com (M.H.)

**Keywords:** rAAV, ITRs, productivity, potency, packaging, nanopore sequencing

## Abstract

One of the primary challenges in working with adeno-associated virus (AAV) lies in the inherent instability of its inverted terminal repeats (ITRs), which play vital roles in AAV replication, encapsidation, and genome integration. ITRs contain a high GC content and palindromic structure, which occasionally results in truncations and mutations during plasmid amplification in bacterial cells. However, there is no thorough study on how these alterations in ITRs impact the ultimate AAV vector characteristics. To close this gap, we designed ITRs with common variations, including a single B, C, or D region deletion at one end, and dual deletions at both ends of the vector genome. These engineered ITR-carrying plasmids were utilized to generate AAV vectors in HEK293 cells. The crude and purified AAV samples were collected and analyzed for yield, capsid DNA-filled percentage, potency, and ITR integrity. The results show that a single deletion had minor impact on AAV productivity, packaging efficiency, and in vivo potency. However, deletions on both ends, except A, showed significant negative effects on the above characteristics. Our work revealed the role of ITR regions, A, B, C, and D for AAV production and DNA replication, and proposes a new strategy for the quality control of ITR-bearing plasmids and final AAV products.

## 1. Introduction

The *adeno-associated virus* (AAV) is a small, non-enveloped virus belonging to the *Parvoviridae* family. AAV is of significant interest to researchers and clinicians due to its capacity to serve as a versatile and safe vector for delivering genetic material into a wide range of host cells. This remarkable property has propelled AAV into the spotlight of gene therapy, where it plays a pivotal role in the development of promising treatments for various genetic and acquired diseases [1]. The AAV particle has a simple and elegant structure, comprising a small, single-stranded DNA genome encased within a protein capsid. One of the distinctive features of the AAV genome is the presence of inverted terminal repeats (ITRs), which are identical sequences found at both ends of the DNA strand [2]. ITRs are the only genetic elements required in cis to produce recombinant AAV vectors [3]. They flank AAV genomes and play important roles in replication, encapsidation, and integration into the host genome [4]. Each ITR consists of 145 nucleotides. The terminal 125 nucleotides (nts) form a T-shaped hairpin structure by self-annealing. The hairpin structure has two palindromic arms, B-B′ (B loop) and C-C′ (C loop), and one stem formed by the palindromic A-A′ [5]. The first inbound 20 nts are the only non-palindromic region, which is known as the D sequence. The ITR also contains a Rep binding element (RBE) and a terminal resolution site (TRS). There are two orientations of ITR, which are flip and flop. Flip has the B loop closer to the 3′ end whereas flop has the C loop closer to the 3′ end [5]. The AAV genome can be flanked by two flip-oriented domains, two flop-oriented domains, or one ITR of each orientation [6]

ITR sequences have been extensively modified to understand their structure–function relationships and improve AAV-based gene therapy vectors. There are two main categories of modifications, which are a directed structure modification and combination of ITRs from different serotypes [7]. The directed structure modification involves mutating or deleting specific regions of ITR. Truncation of terminal 14 bp at both ITR [6] or 11 bp in 3′ITR has been demonstrated to have no impact on AAV production [8]. B and C loops are found to not be required for encapsulation but essential for vector genome replication. The deletions of both B and C loops in two ITRs resulted in higher potency in vitro and in vivo [9]. The D sequence has been shown to be important for genome encapsulation [10]. Mutagenesis in the RBE site leads to a diminished binding affinity for Rep proteins [11]. Mutagenesis in the TRS site reduces Rep nicking [12]. ITRs that are completely free of CpG motifs have been investigated to reduce immune responses to gene therapy, while these CpG-free ITRs may result in lower vector yields, they maintain genome encapsidation levels and offer potential advantages in reducing immune-related complication [13].

A combination of ITRs from different AAV serotypes has been explored to optimize AAV vectors. Although the ITR of AAV2 is the most widely used and compatible with various serotypes, it may not always be the optimal ITR. So, hybrid ITRs have been developed. AAV2-5 ITR showed increased efficiency in gene expression compared to vectors with AAV2 ITRs [14]. In the context of AAV serotype 3 production, the pairing of Rep3 with ITR3 was found to enhance viral titer and boost transduction efficiency in human hepatocellular carcinoma cell lines in vitro, resulting in a 4-fold improvement compared to Rep2 and ITR2 [15]. These modifications have demonstrated the impact of the ITR sequence in the biology of AAV.

ITRs are recognized for their inherent instability [5,16,17], mainly due to their palindromic configuration and heightened GC content. These characteristics promote the occurrence of truncations and mutations when ITR-bearing plasmids are amplified within bacterial cells. The most frequently observed alteration occurs as a small deletion in the B or C loop of one of the ITRs. These small deletions are usually considered non-problematic because the mutated ITR has the unique ability to self-repair during the AAV replication process, probably via the intact ITR at the other end of the genome [18,19]. However, the repair mechanism interferes with elucidating the functional role of the B and C loops. To comprehensively investigate the repercussions of deleted ITRs on AAV production yields, DNA packaging efficiency, potency, and genome integrity, we employed flop-oriented AAV2 ITRs to design a series of plasmids featuring truncations in the B loop, C loop, B-C loop, A region, or D sequence. These deletions were intentionally incorporated into a single ITR as well as both ITRs. We draw comparisons between AAV vectors generated using these engineered plasmids and those containing unaltered, wild-type ITRs. This analysis is aimed at shedding light on the impact of deleted ITRs on recombinant AAV characteristics throughout the manufacturing process to potency.

## 2. Materials and Methods

### 2.1. Plasmid Design and Construction

Plasmids have deletion in either one ITR or both ITRs. The WT ITR at both ends is used as the control. The plasmids with truncated ITRs were synthesized and prepared by GenScript. The sequences described below represent the 3′ ITR of an AAV genome.

Wild-type ITR sequence:

5′-aggaacccctagtgatggagttggccactccctctctgcgcgctcgctcgctcactgaggccgggcgaccaaaggtcgcccgacgcccgggctttgcccgggcggcctcagtgagcgagcgagcgcgcagagagggagtggccaa-3′

C loop deleted ITR sequence:

5′-aggaacccctagtgatggagttggccactccctctctgcgcgctcgctcgctcactgaggccgggcgaccaaaggtcgcccggcctcagtgagcgagcgagcgcgcagagagggagtggccaa-3′

B loop deleted ITR sequence:

5′-aggaacccctagtgatggagttggccactccctctctgcgcgctcgctcgctcactgaggccgcccgggctttgcccgggcggcctcagtgagcgagcgagcgcgcagagagggagtggccaa-3′

B and C loop deleted ITR sequence:

5′-aggaacccctagtgatggagttggccactccctctctgcgcgctcgctcgctcactgaggccggcctcagtgagcgagcgagcgcgcagagagggagtggccaa-3′

D region deleted ITR sequence:

5′-tggagttggccactccctctctgcgcgctcgctcgctcactgaggccgggcgaccaaaggtcgcccgacgcccgggctttgcccgggcggcctcagtgagcgagcgagcgcgcagagagggagtggccaa-3′

A region deleted ITR sequence:

5′-aggaacccctagtgatggagttggccactccctctctgcgcgctcgctcgctcactgaggccgggcgaccaaaggtcgcccgacgcccgggctttgcccgggcggcctcagtgagcgagcgagcgcgcag-3′

The transgene cassette, which includes a liver-specific promoter driven human factor IX (*hFIX*), was flanked by ITR sequences. All plasmids were amplified in *E. coli* Stbl3 (C737303, ThermoFisher, Waltham, MA, USA) strain.

### 2.2. AAV Production

HEK293F (ThermoFisher, Waltham, MA, USA) cells were cultivated in 2.8 L shake flasks to prepare for vector production. Prior to transfection, cell counts were initially assessed using the Vi-Cell XR Cell Counter (Beckman Coulter, Pasadena, CA, USA) to ensure that viable cell concentrations fell within the range of 2.0 × 10^6^ to 2.6 × 10^6^ cells/mL, with viabilities exceeding 95%. The respective mass proportions of pHelper, pRep/Cap, and pGOI were 0.43, 0.35, and 0.22. The capsid variant was AAV-DJ [20], and the gene of interest (GOI) was human *FIX* driven by a liver-specific promoter. The transfection reagent employed was FectoVIR (101000004, Polyplus, SA, France). Cell harvesting was performed 72 h following transfection of the cultures. The harvested cells underwent treatment with a lysis buffer (1× PBS, 1 mM MgCl_2_, 0.5% Triton-x 100 (ThermoFisher, Waltham, MA, USA)) to release AAV particles, followed by the removal of cellular debris using a high-speed centrifuge. Subsequently, purification was carried out utilizing ÄKTA pure 25 (Cytiva, Malborough, MA, USA) to isolate AAV particles from cellular debris and impurities. The concentrated AAV underwent an additional step of CsCl ultracentrifugation to eliminate empty particles. The ultimate AAV product was then formulated in a suitable buffer and stored in a −80 °C freezer for preservation.

### 2.3. AAV Titration by Droplet Digital PCR (ddPCR)

In the process of AAV titration, the ultimate product underwent an initial treatment with Dnase I to eliminate any potential host cell DNA and plasmid DNA, then subjected to proteinase K treatment to extract DNA from the capsids. Afterward, the samples were diluted and combined with a ddPCR master mix that included a primer/probe set designed to specifically bind to the GOI cassette. The primer/probe set used had the following sequences: forward primer: 5′-ctccaccttggacacaggac-3′, reverse primer: 5′-ggcagtgtacagcttccact-3′, probe: 5′-gctgtggtttctgagccaggtaca-3′, with a focus on the liver-specific promoter sequence. To create droplets for each sample, a Bio-Rad Automated Droplet Generator (Bio-Rad, Hercules, CA, USA) was employed, and these droplets were subsequently subjected to thermocycling to amplify the DNA of interest through standard PCR procedures. Both positive and negative droplets were quantified using the Bio Rad QX200 Droplet Reader (Bio-Rad, Hercules, CA, USA) and analyzed using Poisson distribution analysis. The resulting amplicon copy numbers were adjusted by sample preparation to provide a concentration measurement in units of copies per milliliter (copies/mL).

### 2.4. AAV Purification by Solid-Phase Extraction (SPE)

The purification of AAV samples was accomplished through a Solid-Phase Extraction (SPE) method. The process is based on capsid binding using affinity resin in a 96-well plate. The Pall Vacuum Manifold (16003-836, Cytiva, Malborough, MA, USA) and Analog Rocking Platform Shaker were instrumental in efficiently executing the purification procedure. The POROS Capture Select AAVX Affinity Resin (A36741, ThermoFisher, Waltham, MA, USA) was distributed within a Pall Acro prep 96-well filter plate. The Pall Vacuum Manifold and Analog Rocking Platform Shaker were utilized for the incubation, washing, and elution steps. The details for this method can be found in the user guide for Capture Select™ AAVX Affinity Resin from the Thermo Fisher Scientific website.

### 2.5. Empty/Full Capsid Measurement by High-Performance Liquid Chromatography (HPLC)

The full and empty ratio of produced AAV samples was assessed via High-Performance Liquid Chromatography (Vanquish Flex UHPLC, ThermoFisher, Waltham, MA, USA). The AAV samples, SPE purified, were injected into an HPLC system equipped with a size-exclusion chromatography (SEC) column, chosen for its ability to separate AAV particles based on size. Calibration standards with known empty/full ratios were injected to generate a standard curve. Detection was carried out using UV absorbance (Variable Wavelength Detector F, VF-D40-A, ThermoFisher, Waltham, MA, USA). Empty and full capsids eluted at the same time on this column. The A260:A230 ratio was used to measure empty/full capsid (Chromeleon 7.3 Chromatography Data System Software).

### 2.6. AAV DNA Checking by Nanopore Sequencing

This procedure encompassed both the purification and library preparation of DNA samples. In a 200 µL PCR tube, 100 µL of sample and 50 µL of HL-SAN mixture (Salt Active Nuclease) were combined and incubated for 30 min at 37 °C, followed by rapid cooling on ice. After the addition of EDTA, the processed sample was combined with PB Buffer (QIAquick PCR Purification Kit (50), Qiagen, Hilden, Germany) and subjected to further incubation. A pH indicator was introduced; in this process, the sample should exhibit a yellow color; if it appears orange, sodium acetate is included. Subsequently, the sample was loaded onto a QIAquick column, followed by washing and elution. Post-elution, the DNA underwent dilution, fragmentation, and addition of adapter sequences. The Rapid Barcoding kit (SQK-RBK004, Oxford Nanopore Technologies, Oxford, UK) was used to generate barcoded sequencing libraries. In this process, the transposase simultaneously cleaves template molecules and attaches barcoded tags to the cleaved ends. Subsequently, the barcoded samples were pooled, and rapid sequencing adapters were added to the tagged ends. The library was then loaded onto a MinION Mk1B flow cell and sequencing commences, continuing until enough reads were generated. Finally, base calling and sequence alignment procedures were performed with minimap2, and the alignment outcomes were evaluated for genome coverage, structural soundness, and potential variations were visualized with Geneious Prime. Essential quality metrics such as read accuracy, sequencing depth, and coverage uniformity were computed to gauge AAV vector sequence fidelity.

### 2.7. In Vitro Potency

Potency of AAV-DJ vectors with different designed ITRs were carried out by measuring human factor IX (hFIX) expression in vitro. Huh7 cells were cultivated in Dulbecco’s Modified Eagle Medium (Life Technologies, Grand Island, NY, USA) supplemented with 10% Fetal Bovine Serum (Life Technologies, Grand Island, NY, USA) and 1% penicillin–streptomycin (Life Technologies, Grand Island, NY, USA) and were seeded at a density of 5 × 10^4^ cells per well in 48-well plates. Purified AAV-DJ vectors, possessing distinct ITR designs, were transduced at varying multiplicities of infection (MOI) levels: 1000, 4000, 16,000, 64,000, and 256,000 viral particles per cell. Culture media were collected and analyzed for hFIX concentrations by an enzyme-linked immunosorbent assay (ELISA), 72 h after transduction. For the hFIX assay, a mouse monoclonal anti-hFIX antibody (Sigma-Aldrich, St. Louis, MO, USA) was used as the capture antibody at the concentration of 4 µg/mL, and a polyclonal goat anti-hFIX antibody coupled to horseradish peroxidase (Affinity Biologicals, Hamilton, ON, Canada) was utilized as the detection antibody at the concentration of 0.1 µg/mL. Samples were diluted in a sample dilution buffer (1% bovine serum albumin/1% tween20/phosphate-buffered saline) to concentrations within the range of the standard curve. The hFIX standard protein (Thermo Fisher, Waltham, MA, USA) was used for standard curve preparation with the range from 0.069 ng/mL to 50 ng/mL. The inter-assay CVs were in the range of 2.8–4.7%, and the lowest detection level was 0.01 ng/mL. Statistical analysis was applied to discern differences in protein expression across the MOI groups, facilitating a comprehensive evaluation of the AAV-DJ vector’s in vitro potency in terms of its transgene protein production capabilities.

### 2.8. AAV Genome Replication for Dual Deletion Design with ddPCR

To assess the replication capability of GOI plasmids with altered ITRs, we transfected the plasmids in triplicate into the suspension AAV production cell line. A pAd-helper-Rep plasmid (without cap gene) was used to assess the DNA replication in the absence of DNA packaging into capsids. To remove any background signal, we tested the condition without the pAd-helper and Rep-Cap as negative control. Under all the tested conditions, the suspension cells with media were harvested at 72 h post-transfection. Samples containing 5 mL from each test condition were collected and lysed (10× lysis buffer, 10% Tween 20 + 500 mM Tris + 20 mM MgCl_2_ pH = 8.0). The samples were centrifuged at 10,000 g/min, 5 min; the supernatant was collected and submitted for AAV genome copy number analysis by ddPCR.

## 3. Results

### 3.1. Design of ITRs with Different Deletions

**Figure 1:**
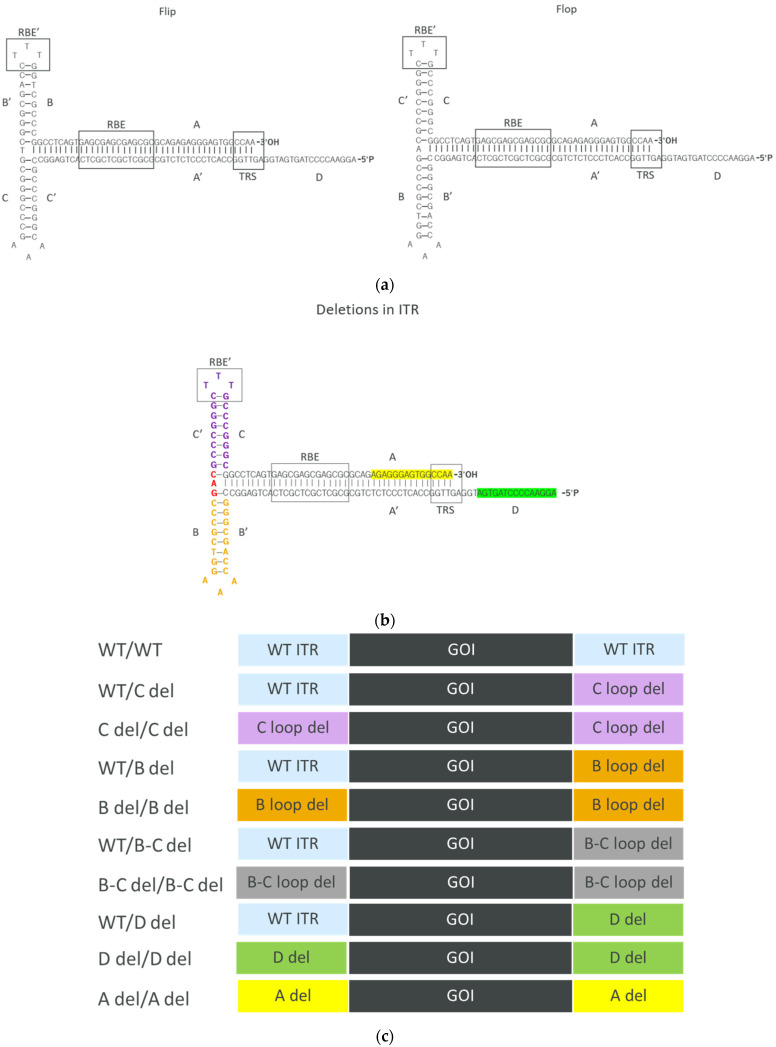
(**a**) WT AAV2 ITR in the flip and flop configurations. In this study, the ITR with flop configuration was utilized. Rep binding element (RBE), the second Rep binding element (RBE’), and the terminal resolution site (TRS) are highlighted in black boxes. (**b**) Highlighted sequences for different deletions in ITRs. B loop deletion: orange and red base pairs. C loop deletion: purple and red base pairs. A region deletion: base pairs highlighted in yellow. D sequence deletion: base pairs highlighted in green. (**c**) Deletions were generated in either one or two of the ITRs. In total, 10 different constructs were created.

The AAV ITR sequence is derived from the work of Srivastava et al. [21] and comprises four major regions: A/A′ stem, B/B′ arm, C/C′ arm, and D sequence. The functionality of the ITR relies on three crucial sequence elements: 16 bp (5′-GAGCGAGCGAGCGCGC) Rep binding element (RBE), 5 bp (5′-CTTTG) second Rep binding element (RBE’), and 6 bp (5′-GGTTGA) terminal resolution site (TRS) [5,22]. To modify the ITRs, we employed the 145 bp AAV2 ITR as the wild-type (WT) template and generated deletions based on it. Two configurations of ITRs exist, namely “flip” and “flop”, with the flop configuration (Figure 1a) being utilized in this study. To investigate the impact of ITR deletions, we designed several constructs with deletions in one or two ITRs focusing on the B loop, C loop, D sequence, and A region. The different deletions in the ITRs can be found in Figure 1b. Ten constructs with different deletions in the ITRs were created for further evaluation (Figure 1c).

**Figure 2:**
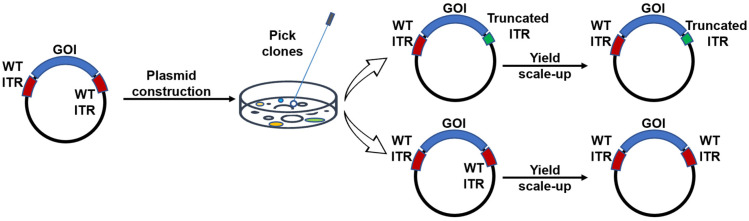
Mutations were identified in the ITRs during the plasmid construction phase. Initially, a plasmid containing intact 145 bp ITRs was utilized. However, following digestion and subsequent construct generation, approximately 40% of the colonies exhibited mutations within the ITRs. Notably, these ITR mutations were observed exclusively in one of the ITRs. Upon colony selection, the sequence of the ITRs was preserved throughout the plasmid expansion process.

ITRs are known to be GC rich but are inherently unstable. Accordingly, in our experiments using stbl3-competent cells for transformation, we observed that ~40% colonies of the wild-type ITR constructs had spontaneous mutations in the ITRs during the cloning process. Notably, these mutations exclusively occurred in one of the ITRs, specifically with either a C loop or B loop deletion. This observation was consistent with previous published results [5,16]. Interestingly, after the construct with wild-type (WT) ITRs was selected, no further mutations were observed during the plasmid expansion process. Moreover, in the constructs bearing only one engineered ITR, no spontaneous mutations were observed in the WT ITR at the other end of the vector genome (Figure 2).

### 3.2. Productivity of Vectors with ITRs Possessing Different Deletions

To gauge AAV productivity, we transfected suspension HEK293F cells with the Rep-Cap plasmid, Helper plasmid, and one of the 10 ITR-containing plasmids described above. Harvesting was performed 72 h post-transfection. A 5 mL sample was treated with lysis buffer, and benzonase was added to degrade plasmid DNA, and then the packaged vector genomes were released from the capsids using proteinase K. The quantification of the vector genome copy number was accomplished using droplet digital PCR (ddPCR) with primers/probe targeting the liver-specific promoter 1 (LSP1) sequence shared by all vectors.

**Figure 3:**
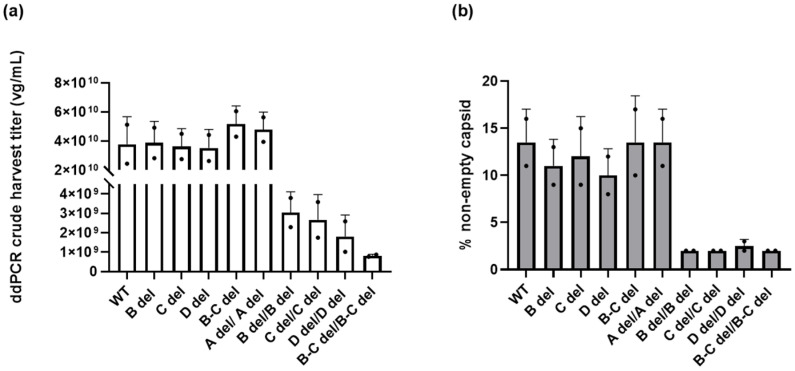
The impact of deletions within the ITRs on crude harvest titer and percentage of non-empty capsids (%). (**a**) Vector genome titration in the crude harvest using ddPCR. (**b**) Measurement of the DNA-filled capsid percentage (non-empty capsids) using SEC HPLC. The error bars represent standard deviation of the mean (*n* = 2).

Figure 3a summarizes the impact of ITR deletions on crude harvest titers. The crude harvest titer of WT/WT was determined to be 3.79 ± 1.90 × 10^10^ vg/mL. The titers of vectors with A del/A del, WT/B del, WT/C del, WT/D del, and WT/B-C del ranged from 3.53 ± 1.27 × 10^10^ vg/mL to 5.185 ± 1.24 × 10^10^ vg/mL, which were comparable to WT/WT.

However, the titer of vectors with deletions in the C loop, B loop, and D sequence in both ITRs were determined to be 8.19 ± 0.728 × 10^8^ vg/mL to 3.05 ± 1.07 × 10^9^ vg/mL. These vectors exhibited a substantial 12- to 21-fold reduction in crude harvest titer compared to vectors with WT/WT ITRs. Remarkably, when both B loop and C loop were deleted from both ITRs, the vector’s productivity plummeted 46-fold.

### 3.3. Reduced Packaging Efficiency Leads to Diminished Yield

To gain a deeper understanding into the causes responsible for the decreased yield observed in AAV vectors featuring truncated ITRs, we examined the packaging of vector genomes. The crude harvest lysate was purified by solid-phase extraction and vector genome packaging was evaluated by High-Performance Liquid Chromatography (HPLC) as a percentage of non-empty capsids.

As depicted in Figure 3b, our examination of AAV vectors revealed interesting findings when it came to the impact of deletions within the ITRs on vector genome packaging. For vectors containing deletions in the B loop, C loop, or both (B-C) within one of the ITRs, our findings indicate that these modifications did not significantly affect the packaging efficiency of AAV vectors. In fact, the percentage of non-empty capsids in these truncated ITR variants ranged from 11.00 ± 2.83% to 13.50 ± 4.95%, closely resembling that of the vector featuring wild-type ITRs, which was 10.00± 3.54%. Likewise, vectors with deletions in the D sequence exhibited a similar packaging efficiency of 10.00 ± 2.83%. Furthermore, when the A sequence was deleted in both ITRs, the percentage of non-empty capsid is comparable to the vector with wild-type ITRs.

Conversely, when B loop, C loop, D sequence, or B-C loop deletions occur in both ITRs, the percentage of non-empty capsids, 2.50 ± 0.71%, decreased by 5-fold. The reduced crude harvest titer is closely associated with a decreased percentage of non-empty capsids. This suggests that having deletions in both ITRs has a substantial adverse impact on the packaging efficiency of the AAV vectors, except for A region deletions.

### 3.4. Diminished Replication Is a Critical Factor Contributing to the Low Yield in Vectors with Deletions in Both ITRs

In our pursuit to unravel the underlying causes of the reduced yield observed in vectors with deletions in both ITRs, we assessed the genome replication of these vectors in HEK293 by transfection of various ITR-bearing plasmids, in the absence of cap, and with or without rep. To enable this assessment, we generated a special pAd-helper-Rep plasmid, which was co-transfected with GOI plasmids.

**Figure 4:**
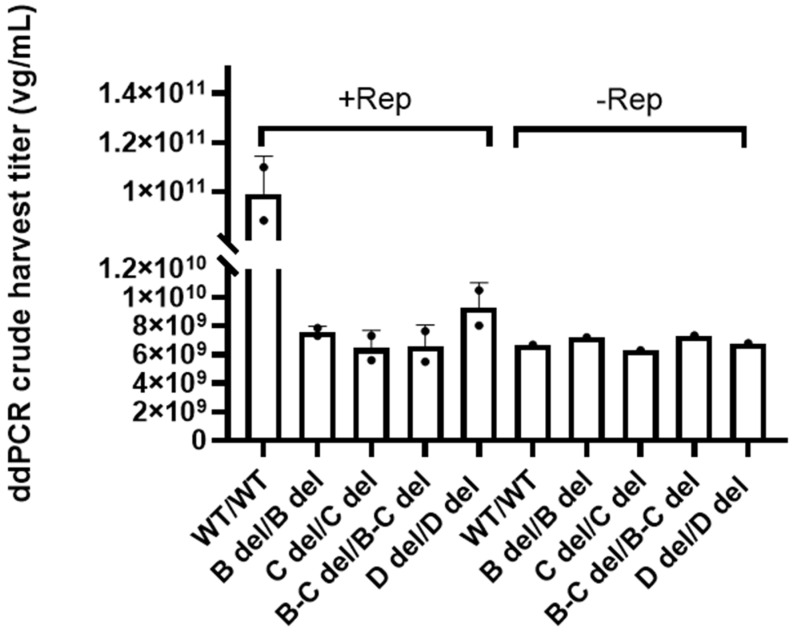
The assessment of AAV genome replication in HEK293 cells with and without Rep. The crude harvest samples were not treated with Benzonase, and the genome copy number was determined by ddPCR. The error bars represent standard deviation of the mean (*n* = 2).

Figure 4 shows that the presence of the Rep gene and wild-type ITRs induced AAV genome replication as evidenced by the increase of vector genomes compared to the control without the Rep gene. The vector copy number was 9.92 ± 1.53 × 10^10^ vg/mL in crude lysate samples. In contrast, the vectors with ITR featuring dual deletions exhibited genome copy numbers decreased by 10- to 16-fold in the crude samples, hovering between 6.46 ± 1.22 × 10^9^ vg/mL and 9.27 ± 1.75 × 10^9^ vg/mL. This level was comparable to the results of the controls without Rep. This substantial reduction clearly underscored the significant impact of dual ITR deletions on AAV genome replication efficiency.

These findings provide crucial insights into the interplay between Rep, ITR, and AAV genome replication, elucidating the intricate factors that influence vector yield.

### 3.5. Assessment of the Packaged DNA Integrity Using Nanopore Sequencing

To evaluate the potential implications of deletions within ITRs on AAV packaging and production, we employed Solid-Phase Extraction (SPE) for the primary purified samples (SPE samples), and Cesium chloride (CsCl) ultracentrifugation for the further purified samples (post-CsCl samples). For both samples, we applied Nanopore sequencing to check the AAV capsid packaged DNA. We mapped the sequencing reads onto the Gene of Interest (GOI)-carrying plasmid and other plasmids that contain DNA sources, such as helper-pAd (LB-Pm-099) and helper-pAAV (LB-Pm-040), as well as the host cell genome, specifically utilizing the GRCh38.p14 as reference genome. This comprehensive analysis for reads encompassed both coverage and attribution considerations.

**Figure 5:**
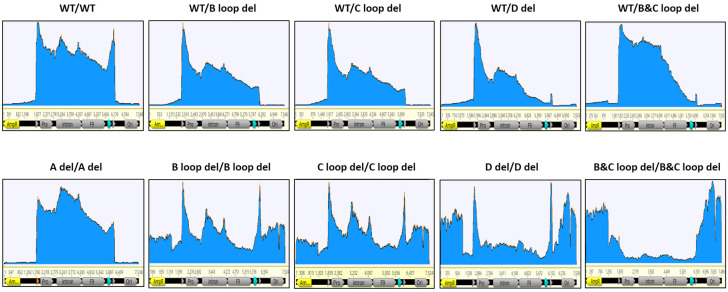
The coverage of nanopore sequencing reads for 10 design constructs (SPE samples). The GOI carrying plasmid was linearized as reference and reads were mapped. Key features are annotated, with the two triangles indicating the ITR regions; the promoter; intron, hFIX, and Poly A (turquoise triangle) are within the two ITRs. AmpR and Ori are in the plasmid backbone region.

For the reads coverage of SPE samples on GOI-bearing plasmid (Figure 5), the vector with wild-type sequence within both ITRs, the majority of reads exhibited alignment to the inter-ITR region, with only a marginal fraction extending beyond this area. Enriched read clusters emerged at both ends of the ITR region. For the GOI region, the sequencing depth displayed positional variation, albeit lacking significant distinctions.

For the B loop deletion in one ITR, the packaged DNA reads also predominantly accumulated within the two ITR regions. However, the enriched read clusters were exclusively concentrated within the intact 5′ ITR region. Remarkably diminished sequencing depth was observed in the 3′ ITR region harboring the C loop deletion, in contrast to the GOI region. Notably, the extent of reads enrichment within the ITR region was lower than that observed in the wild-type design. Analogous outcomes were obtained for other single deletions within the 3′ ITR, such as the C loop deletion in one ITR (referred to as WT/C loop del) and the combination of B and C loop deletions in one ITR (referred to as WT/B and C loop del).

In designs featuring dual identical deletions at both the 5′ and 3′ ITRs, the B loop deletion displayed a substantial influence on plasmid DNA packaging. Numerous reads aligned with the plasmid’s backbone sequence, exhibiting comparable enrichment across both the backbone and GOI sequences. Within the ITR region, the presence of two peaks indicated elevated packaging of ITR copies. A similar distribution of reads was observed for the dual C loop deletion design at both ITRs.

Interestingly, the simultaneous B and C loop deletions at both 5′ and 3′ ITRs yielded a more pronounced impact on DNA packaging than anticipated. A larger proportion of sequencing reads localized outside the two ITR regions. For the GOI DNA itself, packaging was notably reduced compared to the plasmid’s backbone DNA sequence. Even within the ITR sequence, despite not surpassing the count of backbone sequence reads, the number of reads remained substantial enough to preclude the emergence of peaks observed in other design types.

Another distinctive pattern emerged for a single deletion in the 3′ ITR—specifically, the deletion of the D sequence. Notably, the number of mapped reads gradually declined from the 5′ ITR towards an extremely low level at the 3′ ITR. These results underscored that the D sequence deletion significantly curtailed the presence of AAV particles harboring a complete genome.

Furthermore, dual D sequence deletions at both 5′ and 3′ ITRs exerted a significant effect on packaging. Unlike the double B and C loop deletion scenario, these deletions failed to exhibit packaging preferences between the GOI and plasmid backbone sequences. Nevertheless, the ITR regions consistently garnered the highest read counts.

For the design featuring the deletion of two A sequences at both 5′ and 3′ ITRs, a comparison with the WT ITRs revealed minimal disparities in the coverage pattern of reads. Notably, the peaks within the ITR regions were less pronounced than those observed in WT ITRs.

**Figure 6:**
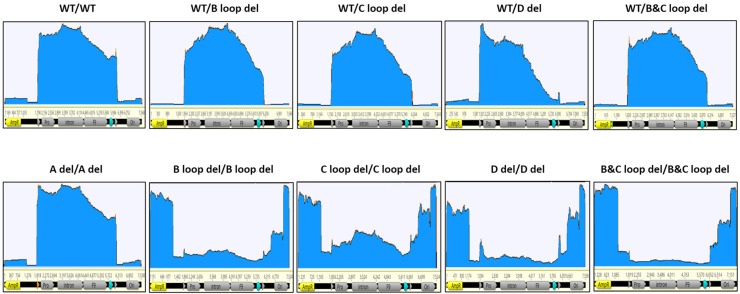
The coverage of nanopore sequencing reads for 10 design constructs (post-CsCl samples). The GOI carrying plasmid was linearized as a reference and reads were mapped. Key features were annotated; the two triangles indicate the ITR regions, the promoter, intron, hFIX, and Poly A (turquoise triangle) are within the two ITRs. AmpR and Ori are in the plasmid backbone region.

To further study the influence of variations in ITRs on the final AAV products, we applied nanopore sequencing to the Cesium chloride (CsCl) ultracentrifugation purified samples. The coverage of reads of post-CsCl samples on GOI-bearing plasmid were generally similar to SPE samples, but the depth of the reads changed (Figure 6).

For all unpurified SPE samples, the reads peaked at one ITR region at least, no matter what kind of deletion was made in the design. However, for post-CsCl samples, there was no obvious reads peak at the ITR region except for “B and C loop del/B and C loop del” and “D del/D del” designs.

To study the source and ratio of the DNA encapsulated in the AAV particles for both SPE and post-CsCl samples, we mapped and quantified the reads to all possible DNA sources.

**Figure 7:**
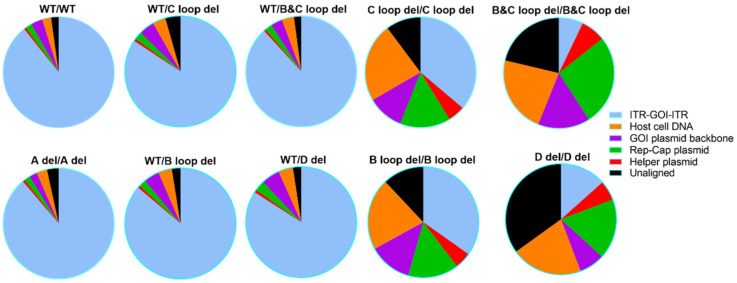
The attribution of Nanopore sequencing reads for 10 designed AAV vectors (SPE samples). The possible sources of packaged DNA are listed: the GOI sequence, the GOI plasmid backbone sequence, the host cell DNA, the Rep-Cap plasmid, and the helper plasmid. The sequencing reads that could not be mapped to any of the above categories were grouped into the unaligned category.

For the SPE samples (Figure 7 and Table S1), we detected DNA from all the possible DNA sources. Among different ITR designs, the DNA category ratio varied a lot. For example, the WT/WT SPE samples showed 89.20% reads attributed to ITR-GOI-ITR region. For the other four single ITR deletion designs, WT/C loop del, WT/B and C loop del, WT/B loop del, and WT/D del, together with A del/A del, the reads’ category ratios are largely similar to WT/WT, with the majority of reads (above 84.10%) attributed to the ITR-GOI-ITR region.

Obvious differences were observed for SPE samples with two deletion designs, those being C loop del/C loop del, B and C loop del/B, and C loop del, B loop del/B loop del, and D del/D del. The packaged GOI sequence was no more than 36.20%. For the B and C loop del at both ITRs, only 7.1% GOI was shown in all the particles. For these vectors, the majority of the packaged DNA sequence comes from the host cell genome and packaging plasmids.

**Figure 8:**
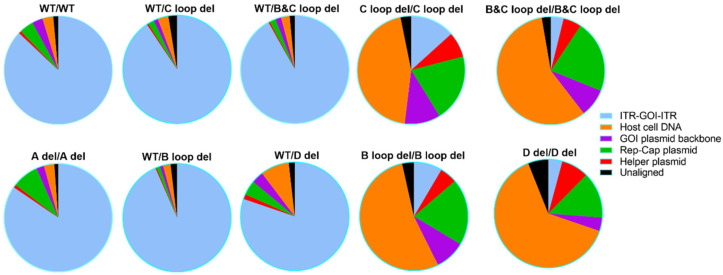
The attribution of Nanopore sequencing reads for 10 designed AAV vectors (post-CsCl samples).

We also analyzed the post-CsCl samples, in general, for WT/WT, A del/A del, and all the other four single deletion designs. The ratios of packaged DNA from different sources are at similar level to corresponding SPE samples (Figure 8 and Table S2). More than 80% of the packaged DNA is transgene (Table S2). This is supported by only one band with the expected size observed on the alkaline gel, except for D del (Figure S1). For the other four dual deletion designs, the ratios for the GOI sequence were further reduced, and ratios for helper plasmid DNA were increased to more than 44.89% when compared to SPE samples. These vectors showed more than one band on the alkaline gel. This finding is consistent with the sequencing results, further supporting the notion that additional genetic material can be packaged in the vector with deletions in both ITRs (Figure S1).

### 3.6. Assessment for the Biological Potency of the Vectors with Modified ITRs In Vitro

In our study, we aimed to investigate the impact of truncated ITRs on the in vitro transduction efficiency of AAV vectors. To do this, we generated AAV vectors with specific ITR deletions, as illustrated in Figure 1. These vectors were then subjected to a transduction experiment using Huh7 cells, with varying Multiplicity of Infection (MOI) levels, ranging from 1000 to 25,600 vg/cell. After 72 h of transduction, we collected the cell culture supernatant for quantification of hFIX expression.

**Figure 9:**
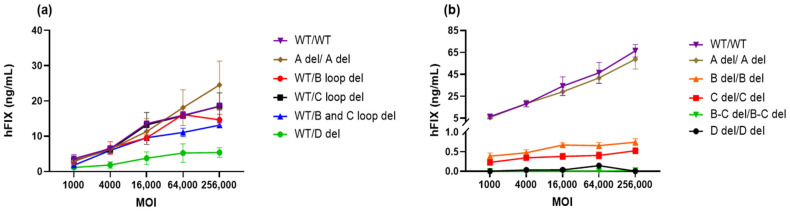
In vitro potency of AAV-DJ-hFIX vectors. (**a**) The expression of hFIX at a series of MOIs ranging from 1000 to 25,600 vg/cell in Huh7 cells was quantified by ELISA and is shown as ng/mL per 1 × 10^5^ cells. (**b**) Purified vectors with deletions in both ITRs were compared to those with wild-type ITRs.

It is worth noting that we employed two different purification methods for the AAV vectors as shown in Figure 9a (SPE) and Figure 9b (affinity chromatography followed by cesium chloride ultracentrifugation). Due to the low percentage of non-empty capsids of vectors with deletions in both ITRs, with the exception of the A region deletions, we implemented cesium chloride ultracentrifugation to selectively eliminate the empty capsid band. This step aimed to enhance the proportion of non-empty capsids in the AAV vectors, thus potentially increasing their effectiveness.

Upon transfecting Huh7 cells with different MOIs of these AAV vectors expressing hFIX, we quantified hFIX expression using ELISA and expressed the results as ng per mL of supernatant. Surprisingly, we observed that the levels of hFIX expression in cells infected with AAV vectors containing B loop, C loop, or B-C loop deletions in one ITR were similar to those observed with the WT vector across all MOIs (Figure 9a). This suggests that these deletions do not significantly impact transduction efficiency.

When AAV-DJ-hFIX vectors were generated from a GOI plasmid with an A region deletion in both ITRs, there was no remarkable difference in potency at any MOIs, except for the highest MOI of 25,600 vg/mL (Figure 9a). However, we observed a reduction in potency in cells infected with vectors carrying a D deletion in one ITR, when compared to the WT ITR. This reduction in potency was particularly evident at MOIs ranging from 4000 to 25,600 vg/cell. A one-way analysis of variance (ANOVA) was conducted to assess the significance of the difference between WT/WT (11.62 ± 6.31 ng/mL) and modified ITRs ranged from 12.67 ± 8.75 ng/mL to 3.48 ± 1.93 ng/mL, at the highest MOI (Figure 9a). Only WT/D del was found to be significant, * *p* < 0.05, which indicates that the D sequence plays a crucial role in the transduction process.

In our comparative analysis between purified vectors containing deletions in both ITRs and those with wild-type ITRs, a striking observation emerged. A del/A del demonstrated a potency closely resembling that of wt/wt in all MOIs, as shown in Figure 9b. Conversely, when we examined the other vectors with various ITR deletions in both ITRs, they consistently displayed lower levels of hFIX expression across all MOIs, with a significant difference determined by ANOVIA (**** *p* < 0.0001). This outcome underscored the critical importance of maintaining the ITR integrity for efficiency of transduction, highlighting the complex interplay between ITRs and other vector components in mediating successful gene delivery.

## 4. Discussion

### 4.1. ITR Function in rAAV and Influence of Potential Truncations

The ITR is a critical component within the AAV vector, and its presence in cis is mandatory for AAV replication, encapsidation, and successful transduction [23,24]. While there are various ITRs derived from different serotypes of AAV, AAV2 is commonly employed in the production and transgene expression of rAAV due to its superior performance. Its core elements have been the subject of extensive study through mutagenesis [6,9].

To further explore the role of ITRs, we introduced truncations in the B, C, or D region of one ITR and both ITRs. It is important to note that ITRs are known to exhibit inherent instability in plasmid preparations, often leading to spontaneous truncations. The instability stems from the high GC content, which typically constitutes around 70% of the ITR sequence, along with their complex palindromic structure [17]. These characteristics of ITRs pose challenges to sequencing them. The complexity and propensity for spontaneous deletions underscore the need for stringent quality control when working with AAV ITRs in recombinant DNA constructs.

In this study, we conducted a comprehensive investigation into the effects of truncated ITRs on the qualities of rAAV. Our findings revealed some intriguing insights. Specifically, we observed that the deletion in one ITR had no discernible adverse effects on various aspects of rAAV. The vectors of WT/B del, WT/C del, and WT/D del WT/B-C del showed similar crude harvest titer, percentage of non-empty capsid, genome packaging, and in vitro potency (Figure 3, Figure 5, Figure 6, Figure 7, Figure 8 and Figure 9). However, when deletions are present in both ITRs, except the A region deletion, we observed reductions in key parameters. These included reduced yield, a lower percentage of non-empty capsids, less-efficient transduction, and increased non-transgene packaging (Figure 3, Figure 4, Figure 5, Figure 6, Figure 7, Figure 8 and Figure 9). These findings suggest the pivotal role of ITRs in maintaining the integrity and functionality of rAAV.

A possible explanation for these findings is the self-repair mechanism of ITR during the second-strand DNA synthesis, which has been proposed by other researchers [16,18,19]. At least one intact ITR is essential for AAV production and transduction. This necessity was confirmed by our observation of vectors with deletions in both ITRs, which exhibited minimal replication, resulting in lower crude harvest titers and reduced percentages of non-empty capsid. Furthermore, these vectors demonstrated reduced potency compared to vectors with either the WT ITR or A region deletion. These results collectively indicate that when both ITRs are compromised, there is a clear adverse effect on ITR function, subsequently influencing the overall quality of rAAV.

Among the 10 vectors featuring various deletions in the ITRs, the A region deletion in both ITRs is noteworthy. It not only exhibits a comparable crude harvest titer, percentage of non-empty capsids, genome packaging efficiency, and in vitro potency in comparison to the WT ITR, but it also shows no mutations during the plasmid construction process. This suggests that the A deletion may be more stable than the WT ITR. It is worth noting that the vectors with A region deletions appear to be very stable. While vectors with deletions in one of their ITRs may undergo a self-repair mechanism during AAV replication, the A deletion appears to be compensated for through the A’ for AAV replication. This compensation mechanism provides a plausible explanation for why the A deletion can closely match the performance of the wild-type ITR, as it adapts to fulfill the necessary functions for AAV replication.

### 4.2. NGS for Checking Plasmid and Vector Homogeneity

Both ITR-carrying plasmids and rAAV vectors should not be treated as homogeneous products, in particular, there is heterogeneity in the double-stranded plasmid DNA and the single-stranded AAV genomic DNA. Especially, the purity of these plasmids, to a large extent, is a major determinant in the purity and quality of the final rAAV product. Contamination caused by heterogeneity in rAAV vector preparations has always been a major issue in gene therapy [25]. However, in assessing the quality of plasmids and rAAV vectors, the attention is frequently directed toward factors other than their homogeneity. For instance, when evaluating plasmids, the prioritized considerations usually are the accuracy of the sequence construct, the impact of the solvent on transfection efficiency, and the level of endotoxins present. In the case of rAAV products, different assays are conducted to determine the titer, capsid protein ratio, the size of the packaged AAV genome, and the presence of DNA from other sources [26,27].

The primary obstacle to the detection of ITR-containing plasmids and rAAV genomes stems from the complex nature of the ITR sequence, characterized by an exceptionally high GC base content (70%) and its palindromic structure. Over the past few decades, the limitations of sequencing technology have presented challenges in acquiring precise ITR sequence data. Even in the present day, utilizing Sanger sequencing for AAV ITR remains not only time intensive but also relatively costly [28,29,30]. Nevertheless, in recent years, the advancement of third-generation sequencing, notably exemplified by Nanopore and Pacbio sequencing technologies, has made it feasible to identify longer sequences with remarkable accuracy. Significantly, these technologies are adept at discerning intricate ITR structures, opening up the potential for us to characterize the homogeneity of plasmid and rAAV genome sequences [31]. Moreover, as the third-generation sequencing library is derived from all potential DNA sources, enclosed within the rAAV capsid, a single sequencing process yields comprehensive information about various DNA sequences, including host DNA that is packaged, the DNA of the packaging plasmid backbone, and incompletely replicated rAAV genome DNA [16,30,32]. Furthermore, other research has indicated that specific complex segments within the rAAV genome sequence might serve as possible covert resolution sites, potentially resulting in truncation during replication or packaging [33].

In this study, we utilized the Oxford Nanopore MinION sequencer to analyze our rAAV samples. Not only did we get individual DNA packaged information for each sample (Figure 5, Figure 6, Figure 7 and Figure 8), but we also gained insights into the homogeneity of the vectors by comparing the sequence results across vector samples and batches. For the majority of SPE samples, it was apparent that peaks of reads were concentrated within the intact ITR region. This not only validates the ITR’s role as a replication origin but also underscores the presence of an abundance of short fragments within unpurified SPE samples. In the case of dual deletions in both ITRs, the reads were dispersed throughout the entire GOI plasmid, in stark contrast to the WT ITR sample data. Together with the titer information we collected by ddPCR (Figure 3), we observed that dual deletion at both ITRs, except dual A region deletions, essentially hinders the replication of the rAAV genome. The subsequent genome duplication experiments validated this hypothesis (Figure 4).

Furthermore, Nanopore sequencing results accurately reflected the purification efficiency of our AAV production process. Results from CsCl-purified rAAV samples demonstrated a marked reduction in sharp read peaks compared to the SPE samples, indicating the removal of empty and partially filled capsids through ultracentrifugation. For samples with dual deletion in both ITRs, except dual A deletion, it was evident that the predominant sequence package was the plasmid’s backbone. Based on the sequencing results, we can speculate the vectors’ potency performance. The more enrichment of the GOI region, the greater the potency is expected to be. This observation aligns with our potency results (Figure 9).

In our study here, we exclusively used Nanopore sequencing technology to assess vector genome packaging, and obtained insight into vector quality, which further validates the rigorous purification process needed for rAAV production. Compared to other quality control assays, the NGS method measures multiple rAAV characteristics at once.

In summary, the data provided in this study demonstrate that, except for the A region (excluding RBE), deletions in one ITR or the other have a minor impact on rAAV genome replication, packaging, and potency. However, dual deletions in both ITRs have a more pronounced negative effect compared to single deletions. We also employed Nanopore sequencing to analyze all collected rAAV samples, and the results offer valuable insights for various attributes pertaining to rAAV quality. As a result, we conclude that incorporating NGS as a routine quality control method could help improve the optimization of AAV production strategies and enhancement of both vector yield and integrity for preclinical and clinical use.

## Data Availability

Data from this study are available from the corresponding author upon request.

## Supplementary Materials

The following supporting information can be downloaded at: https://www.mdpi.com/article/10.3390/microorganisms12020310/s1, Table S1: The distribution of Nanopore sequencing reads for 10 designed AAV vectors (SPE samples). Table S2: The distribution of Nanopore sequencing reads for 10 designed AAV vectors (Post Cscl samples). Figure S1: Measuring the packaged genome size of 10 designed vectors using alkaline gel.

## References

[1] Naso M.F., Tomkowicz B., Perry W.L., Strohl W.R. (2017). Adeno-Associated Virus (AAV) as a Vector for Gene Therapy. BioDrugs.

[2] Flotte T.R., Berns K.I. (2005). Adeno-associated viral vectors for gene therapy. Lab. Tech. Biochem. Mol. Biol..

[3] Young S.M., Samulski R.J. (2001). Adeno-associated virus (AAV) site-specific recombination does not require a Rep-dependent origin of replication within the AAV terminal repeat. Proc. Natl. Acad. Sci. USA.

[4] Weitzman M.D., Linden R.M. (2011). Adeno-Associated Virus Biology. Methods in Molecular Biology.

[5] Wilmott P., Lisowski L., Alexander I.E., Logan G.J. (2019). A User’s Guide to the Inverted Terminal Repeats of Adeno-Associated Virus. Hum. Gene Ther. Methods.

[6] Savy A., Dickx Y., Nauwynck L., Bonnin D., Merten O.W., Galibert L. (2017). Impact of Inverted Terminal Repeat Integrity on rAAV8 Production Using the Baculovirus/Sf9 Cells System. Hum. Gene Ther. Methods.

[7] Shitik E.M., Shalik I.K., Yudkin D.V. (2023). AAV-based vector improvements unrelated to capsid protein modification. Front. Med..

[8] Feiner R.C., Teschner J., Teschner K.E., Radukic M.T., Baumann T., Hagen S., Hannappel Y., Biere N., Anselmetti D., Arndt K.M. (2019). rAAV Engineering for Capsid-Protein Enzyme Insertions and Mosaicism Reveals Resilience to Mutational, Structural and Thermal Perturbations. Int. J. Mol. Sci..

[9] Zhou Q., Tian W., Liu C., Lian Z., Dong X., Wu X. (2017). Deletion of the B-B’ and C-C’ regions of inverted terminal repeats reduces rAAV productivity but increases transgene expression. Sci. Rep..

[10] Wang X.S., Qing K., Ponnazhagan S., Srivastava A. (1997). Adeno-associated virus type 2 DNA replication in vivo: Mutation analyses of the D sequence in viral inverted terminal repeats. J. Virol..

[11] Ryan J.H., Zolotukhin S., Muzyczka N. (1996). Sequence requirements for binding of Rep68 to the adeno-associated virus terminal repeats. J. Virol..

[12] Snyder R.O., Im D.S., Ni T., Xiao X., Samulski R.J., Muzyczka N. (1993). Features of the adeno-associated virus origin involved in substrate recognition by the viral Rep protein. J. Virol..

[13] Pan X., Yue Y., Boftsi M., Wasala L.P., Tran N.T., Zhang K., Pintel D.J., Tai P.W. (2022). Rational engineering of a functional CpG-free ITR for AAV gene therapy. Gene Ther..

[14] Yan Z., Zak R., Zhang Y., Engelhardt J.F. (2005). Inverted Terminal Repeat Sequences Are Important for Intermolecular Recombination and Circularization of Adeno-Associated Virus Genomes. J. Virol..

[15] Ling C., Yin Z., Li J., Zhang D., Aslanidi G., Srivastava A. (2016). Strategies to generate high-titer, high-potency recombinant AAV3 serotype vectors. Mol. Ther. Methods Clin. Dev..

[16] Tran N.T., Lecomte E., Saleun S., Namkung S., Robin C., Weber K. (2022). Human and Insect Cell-Produced Recombinant Adeno-Associated Viruses Show Differences in Genome Heterogeneity. Hum. Gene Ther..

[17] Mroske C., Rivera H., Ul-Hasan T., Chatterjee S., Wong K.K. (2011). Naturally Occurring Non-Functional Mutations of the AAV ITR. Mol. Ther..

[18] Samulski R.J., Srivastava A., Berns K.I., Muzyczka N. (1983). Rescue of adeno-associated virus from recombinant plasmids: Gene correction within the terminal repeats of AAV. Cell.

[19] Samulski R.J., Berns K.I., Tan M., Muzyczka N. (1982). Cloning of adeno-associated virus into pBR322: Rescue of intact virus from the recombinant plasmid in human cells. Proc. Natl. Acad. Sci. USA.

[20] Grimm D., Lee J.S., Wang L., Desai T., Akache B., Storm T.A., Kay M.A. (2008). In Vitro and In Vivo Gene Therapy Vector Evolution via Multispecies Interbreeding and Retargeting of Adeno-Associated Viruses. J. Virol..

[21] Srivastava A., Lusby E.W., Berns K.I. (1983). Nucleotide sequence and organization of the adeno-associated virus 2 genome. J. Virol..

[22] Linden R.M., Ward P., Giraud C., Winocour E., Berns K.I. (1996). Site-specific integration by adeno-associated virus. Proc. Natl. Acad. Sci. USA.

[23] Zhu X., Li J., Xiao X. (2004). Absence of T-Shaped Structure and Deletions of B and C Hairpins Have Minimal Effects on Essential Functions of AAV Inverted Terminal Repeats. Mol. Ther..

[24] Earley L.F., Conatser L.M., Lue V.M., Dobbins A.L., Li C., Hirsch M.L., Samulski R.J. (2020). Adeno-Associated Virus Serotype-Specific Inverted Terminal Repeat Sequence Role in Vector Transgene Expression. Hum. Gene Ther..

[25] Wright J.F. (2014). Product-related impurities in clinical-grade recombinant AAV vectors: Characterization and risk assessment. Biomedicines.

[26] Chadeuf G., Ciron C., Moullier P., Salvetti A. (2005). Evidence for encapsidation of prokaryotic sequences during recombinant adeno-associated virus production and their in vivo persistence after vector delivery. Mol. Ther..

[27] Schnödt M., Schmeer M., Kracher B., Krüsemann C., Espinosa L.E., Grünert A. (2016). DNA Minicircle Technology Improves Purity of Adeno-associated Viral Vector Preparations. Mol. Ther. Nucleic Acids.

[28] Lecomte E., Tournaire B., Cogné B., Dupont J.B., Lindenbaum P., Martin-Fontaine M., Broucque F., Robin C., Hebben M., Merten O.-M. (2015). Advanced characterization of DNA molecules in rAAV vector preparations by single-stranded virus next-generation sequencing. Mol. Ther. Nucleic Acids.

[29] Guerin K., Rego M., Bourges D., Ersing I., Haery L., Harten DeMaio K., Sanders E., Tasissa M., Kostman M., Tillgren M. (2020). A Novel Next-Generation Sequencing and Analysis Platform to Assess the Identity of Recombinant Adeno-Associated Viral Preparations from Viral DNA Extracts. Hum. Gene Ther..

[30] Namkung S., Tran N.T., Manokaran S., He R., Su Q., Xie J., Gao G., Tai P.W.L. (2022). Direct ITR-to-ITR Nanopore Sequencing of AAV Vector Genomes. Hum. Gene Ther..

[31] Tai P.W.L., Xie J., Fong K., Seetin M., Heiner C., Su Q., Weiand M., Wilmot D., Zapp M.L., Gao G. (2018). Adeno-associated Virus Genome Population Sequencing Achieves Full Vector Genome Resolution and Reveals Human-Vector Chimeras. Mol. Ther. Methods Clin. Dev..

[32] Radukic M.T., Brandt D., Haak M., Muller K.M., Kalinowski J. (2020). Nanopore sequencing of native adeno-Associated virus (AAV) single-stranded DNA using a transposase-based rapid protocol. NAR Genom. Bioinform..

[33] Zhang J., Chrzanowski M., Frabutt D.A., Lam A.K., Mulcrone P.L., Li L., Konkle B.A., Miao C.H., Xiao W.H. (2023). Cryptic resolution sites in the vector plasmid lead to the heterogeneities in the rAAV vectors. J. Med. Virol..

