# Supplementary material for: A Comprehensive Study of the Effects by Sequence Truncation within Inverted Terminal Repeats (ITRs) on the Productivity, Genome Packaging, and Potency of AAV Vectors
Source: Microorganisms. 2024 Feb 1;12(2):310. doi: 10.3390/microorganisms12020310 (PMC10892565; doi:10.3390/microorganisms12020310)
Supplement: Supplementary file 1 [file microorganisms-12-00310-s001.zip › microorganisms-2803403-supplementary.pdf]

	WT/WT	WT/C loop del	C loop del/C loop del	WT/B loop del	B loop del/B loop del	WT/B&C loop del	B&C loop del/B&C loop del	WT/D del	D del/D del	A del/A del
Transgene	89.2%	84.3%	36.2%	86.2%	34.8%	88.2%	7.1%	84.1%	13.4%	88.7%
LB-Pm-099 (helper)	0.6%	0.7%	5.0%	0.7%	4.8%	0.6%	7.2%	0.8%	5.8%	0.7%
LB-Pm-040 (rep-cap)	1.9%	2.3%	14.8%	2.1%	14.8%	2.1%	26.6%	3.0%	17.6%	1.7%
GOI w transgene removed	3.4%	4.5%	10.6%	4.5%	12.6%	3.4%	15.2%	5.4%	7.4%	2.3%
hg38	2.6%	3.8%	23.2%	4.0%	21.0%	3.6%	22.6%	4.3%	20.8%	3.1%
Unaligned	2.2%	4.4%	10.2%	2.5%	12.0%	2.1%	21.3%	2.4%	34.9%	3.4%
Total	100.0%	100.0%	100.0%	100.0%	100.0%	100.0%	100.0%	100.0%	100.0%	100.0%

Table S1. The distribution of Nanopore sequencing reads for 10 designed AAV vectors (SPE samples)

	WT/WT	WT/C loop del	C loop del/C loop del	WT/B loop del	B loop del/B loop del	WT/B&C loop del	B&C loop del/B&C loop del	WT/D del	D del/D del	A del/A del
Transgene	87.0%	90.6%	13.3%	93.4%	8.4%	91.9%	3.8%	80.2%	4.2%	84.5%
LB-Pm-099 (helper)	0.7%	0.4%	7.6%	0.3%	5.1%	0.4%	5.4%	1.4%	8.1%	0.8%
LB-Pm-040 (rep-cap)	4.2%	1.8%	20.2%	1.1%	19.8%	1.8%	21.7%	4.3%	13.8%	7.9%
GOI w transgene removed	3.2%	1.2%	10.7%	0.9%	9.0%	1.7%	8.4%	3.5%	4.0%	2.3%
hg38	3.2%	3.2%	44.8%	2.2%	53.8%	2.5%	57.7%	8.5%	63.7%	3.1%
Unaligned	1.4%	2.5%	3.1%	1.9%	3.5%	1.5%	2.7%	1.8%	6.0%	1.1%
Total	100.0%	100.0%	100.0%	100.0%	100.0%	100.0%	100.0%	100.0%	100.0%	100.0%

Table S2. The distribution of Nanopore sequencing reads for 10 designed AAV vectors (Post Cscl samples)

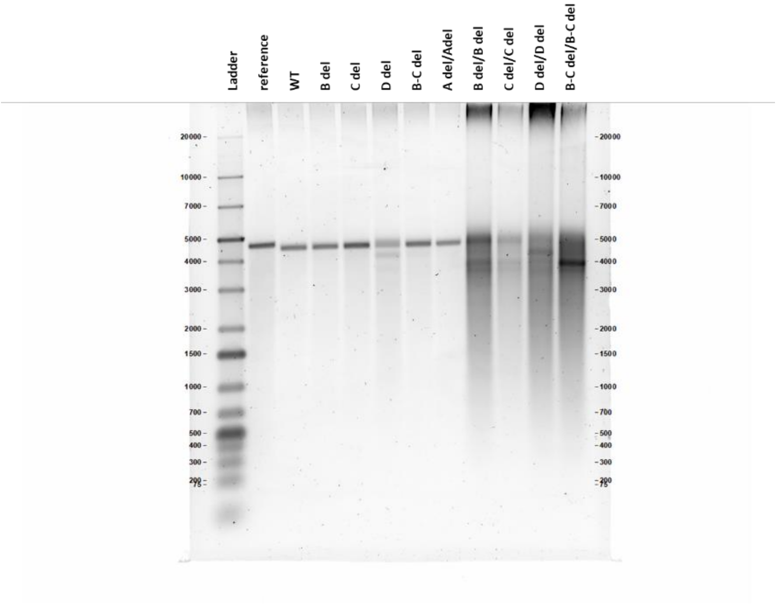

Figure S1. Measuring the packaged genome size of 10 designed vectors using alkaline gel.
